# Weight Trajectory Impacts Risk for 10 Distinct Cardiometabolic Diseases

**DOI:** 10.1210/clinem/dgaf348

**Published:** 2025-06-11

**Authors:** Alison Z Swartz, Kathryn Wood, Eric Farber-Eger, Alexander Petty, Heidi J Silver

**Affiliations:** School of Medicine, Vanderbilt University, Nashville, TN 37232, USA; Department of Medicine, Division of Gastroenterology, Hepatology, and Nutrition, Vanderbilt University Medical Center, Nashville, TN 37232, USA; Department of Pharmacology, Vanderbilt University Medical Center, Nashville, TN 37232, USA; Vanderbilt Genetics Institute, Vanderbilt University Medical Center, Nashville, TN 37232, USA; Department of Medicine, Division of Gastroenterology, Hepatology, and Nutrition, Vanderbilt University Medical Center, Nashville, TN 37232, USA; Department of Veterans Affairs, Tennessee Valley Healthcare System, Nashville, TN 37212, USA

**Keywords:** obesity, weight cycling, weight gain, cardiovascular disease, diabetes

## Abstract

**Context:**

Repetitive bouts of weight loss and regain, termed weight cycling, may exaggerate the risk for cardiometabolic disease. We previously identified that weight cyclers were likely to have prescribed medications for hypertension, dyslipidemia, and diabetes—suggesting a high prevalence of cardiometabolic disease.

**Objective:**

No prior study has compared relationships between longitudinal weight trajectory (weight stable, weight gainer, weight loser, or weight cycler) and commonly occurring specific cardiometabolic diseases among persons with similar high baseline body mass index (BMI).

**Methods:**

Using de-identified electronic health record data from all adults treated at Vanderbilt University Medical Center between 1997 to 2020 and a landmark approach, we developed multivariate Cox proportional hazards regression models to determine relationships between weight trajectory and risk for 10 highly prevalent cardiometabolic diseases.

**Results:**

Compared to weight stability, weight cycling associated with an almost 30% increased risk for obstructive sleep apnea [hazard ratio (HR) 1.28; 95% confidence interval (CI) 1.15-1.42], metabolic dysfunction-associated steatotic liver disease (HR 1.28; 95% CI 1.08-1.51), and type 2 diabetes (HR 1.23; 95% CI 1.10-1.38). Weight cycling also associated with a more than 50% increased risk for heart failure (HR 1.54; 95% CI 1.31-1.82), although both weight gain and weight loss also showed increased risk for heart failure (HR 1.29; 95% CI 1.08-1.55 and HR 1.32; 95% CI 1.10-1.58, respectively).

**Conclusion:**

The relationship between weight cycling and cardiometabolic disease risk was independent of having high baseline BMI, which was similar among weight trajectory groups. The present findings support promoting either weight stability at high BMI or weight loss if able to be maintained to prevent the incidence of a variety of cardiometabolic diseases.

The burden of cardiometabolic diseases associated with having excess body weight continues to escalate worldwide (1). However, weight loss of as little as 5% can reverse many of the adverse cardiometabolic effects of having excess adiposity (2). Yet, weight loss maintenance is uncommon, with only 20% to 56% of adults maintaining clinically significant weight loss over 2 to 5 years (3, 4). Even with the contemporary efficacy of incretin mimetics for inducing substantial weight loss, adherence to these agents is moderate, with <50% of persons continuing treatment beyond 1 year (5). Consequently, repetitive bouts of weight loss and weight regain, termed “weight cycling,” persist, occurring in 27% to 50% of the adult population (4, 6, 7). A serious public health concern is that weight cycling may be associated with an exaggerated risk for adverse health outcomes greater than that occurring from having high body mass (6, 8-12) Notably, 2 recent meta-analyses showed weight cycling increases the risk for hypertension (HTN), cardiovascular disease, type 2 diabetes (T2DM) (13), and all-cause mortality (14).

Weight cycling may impair cardiometabolic health by altering body composition toward a greater proportion of fat vs lean (muscle) mass (15). Such a reduction in lean mass would reduce daily energy expenditure, increasing the risk for future excess weight gain as well as impairing glucose uptake and insulin action in skeletal muscle (16). Indeed, preclinical models of weight cycling show larger adipocyte size, higher fasting glucose levels, and impaired glucose tolerance (17). Further indicating enhanced risk, weight-cycled mice have increased macrophage recruitment and inflammatory cytokine production in adipocytes (18).

Using de-identified electronic health record data, we determined the longitudinal weight trajectory of all adult patients treated at Vanderbilt University Medical Center (VUMC) between 1997 and 2020 (19). We found that 50% of males and 57% of females experienced at least 1 significant weight-cycling episode within a 5-year observation period. Although baseline body mass index (BMI) did not differ when compared to persons with weight stability, weight loss, or weight gain, the weight cyclers were more likely to have prescribed medications for HTN, dyslipidemia, and diabetes—revealing a greater prevalence of cardiometabolic disease.

Interestingly, a study of >9000 adults with elevated low-density lipoprotein cholesterol levels showed that the increased risk for cardiac events and cardiovascular disease mortality that is associated with weight cycling was independent of average baseline body weight and traditional clinical risk factors (20). Nevertheless, several observational cohort studies have not shown greater risk among weight cyclers (7, 21-24). A remaining question, highly relevant to current clinical practice paradigms, is whether having bouts of weight loss and regain is a stronger predictor of poor cardiometabolic health than having chronically high body mass or experiencing weight gain over a prolonged period of time.

No prior study using data from a large regional comprehensive health system has compared longitudinal relationships between weight trajectory category (weight stable, weight gainer, weight loser, or weight cycler) to the variety of commonly occurring cardiometabolic disease states among persons with similar high baseline BMI. The primary aim of the present study is to determine which of the 4 weight trajectory categories is most robustly associated with increased risk for each of 10 highly prevalent cardiometabolic disease states (25, 26). Determining the influence of each weight trajectory type for specific cardiovascular diseases would inform healthcare providers as well as advance weight management practices in the current highly obesogenic environment.

## Methods

### Data Collection

**Figure 1.**
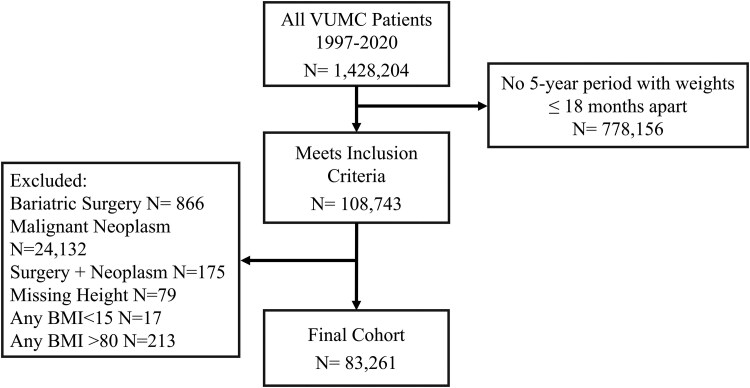
Study eligibility.

Development and cleaning of the dataset has been previously described (19). In brief, data were extracted from a de-identified version (synthetic derivative) of the VUMC electronic health record (EHR) for 1 428 204 adult patients who were treated between 1997 and 2020. Data was included if patients were aged ≥18 years and had a 5-year period of regularly recorded weights with at least 1 weight documented within 18 months of each prior weight. Exclusion criteria included malignant neoplasm (other than nonmelanoma skin cancer), history of bariatric surgery, implausible BMI (<15 or >80 kg/m^2^), weights during pregnancy, or missing height measurement (Fig. 1). The VUMC Institutional Review Board deemed the study exempt (IRB #230831).

### Study Design

**Figure 2.**
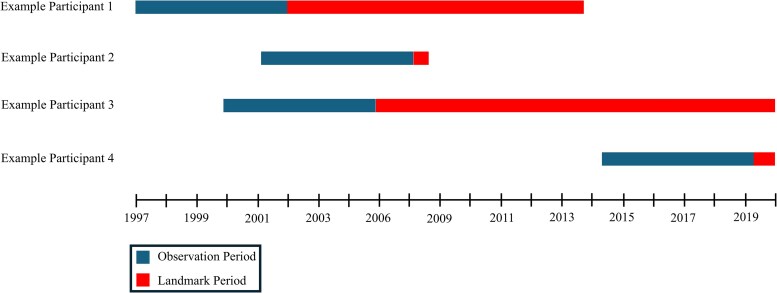
Study design.

A landmark approach, which decreases potential bias in time-to-event analysis (27), was used to identify the occurrence of 10 cardiometabolic disease states [peripheral artery disease, coronary artery disease (CAD), cardiac catheterization, heart failure (HF), myocardial infarction (MI), obstructive sleep apnea (OSA), nonalcoholic fatty liver disease, HTN, hyperlipidemia (HLD), and T2DM] following the initial 5-year observation period. The landmark period ended with the final data extraction from the VUMC synthetic derivative in June 2020 or at the occurrence of a patient's last documentation in the VUMC EHR. Thus, the length of each participant's landmark period depended on the duration of follow-up available in the EHR (Fig. 2). Patients with a diagnosis of 1 or more of the 10 cardiometabolic diseases prior to the onset of the landmark period were excluded from analysis of those outcomes.

### Weight-related Measures

As previously reported, a multistage algorithm was designed to improve the reliability of the weights and heights used in analysis (19). To decrease the impact of multiple recorded weights obtained within a short period of time, linear interpolation was employed to develop a standardized grid of weights with 1 weight every 60 days over the 5-year observation period. Baseline BMI was calculated from median height and first documented weight during the observation period. Using the clinically significant threshold of ≥5% weight change (28-30), weight trajectory was categorized as weight stable, weight gainer, weight loser, or weight cycler. Thus, weight cyclers have at least 1 episode of 5% weight loss and subsequent regain, whereas those with weight stability maintained weights within 5% of each other over the 5-year period.

### Clinical Biomarkers

Age at the beginning of the 5-year observation period was calculated from the date of birth in the EHR. Sex, self-reported race and ethnicity, and smoking status were extracted from the EHR. Smoking status was determined by self-reported documentation or International Classification of Diseases, Ninth Revision (ICD-9) code and categorized as “ever smoker,” “never smoker,” or “unknown.” The earliest documentation of any antihypertensive, antidiabetic, and/or dyslipidemia therapy was extracted from the EHR using MedEX (31). Supplementary Table S1 presents a list of all medications included. Clinical biomarkers by weight trajectory group have been presented in our prior publication (19).

### Cardiometabolic Disease States

Participants were considered to have peripheral artery disease (PAD) if the EHR contained 2 or more PAD ICD-9/International Classification of Diseases, Tenth Revision (ICD-10) diagnosis codes (Supplementary Table S2); 1 PAD ICD 9/10 diagnosis code, which was the primary diagnosis for any in- or outpatient visit; and/or 1 PAD ICD 9/10 diagnosis code coupled with documentation of 1 or more lower extremity revascularization codes, amputation codes, or 2 ankle-brachial-indices performed within 1 year. CAD was defined using a random forest machine learning classifier developed from a combination of ICD-9 and Current Procedural Terminology (CPT) codes, laboratory values, and clinical documentation (32) with the final model having 0.99 specificity for CAD.

HF was defined using eMERGE guidelines (33) and included HF with preserved ejection fraction and reduced ejection fraction. In this cohort, the algorithm for HF was updated to include ICD-10 code I50, and only definite and probable cases were considered. MI was defined as the presence of at least 2 MI-related ICD-9/10 codes less than 5 days apart with at least 1 troponin test, revascularization-related CPT code, or cardiac catheterization-related CPT code within 5 days.

OSA was based on OSA-related ICD-9/10 diagnosis codes. Metabolic dysfunction-associated steatotic liver disease (MASLD) was identified based on the presence of at least 1 MASLD-related reference in clinical notes and at least 1 ICD-9/10 code (Supplementary Table S3). Participants were considered not to have MASLD if they had any medication or diagnosis code indicating an alternate etiology of liver disease, including diagnosed alcohol use disorder, viral hepatitis, and/or medications with a high risk for inducing hepatitis. Of note, although we use current terminology, the data acquired for the present study predate the change in nomenclature from nonalcoholic fatty liver disease to MASLD (34).

HTN was defined by the presence of ICD-9/10 diagnosis codes and problem list documentation of HTN or high blood pressure. HLD was determined by meeting clinical cut-points for low high-density lipoprotein cholesterol, high low-density lipoprotein cholesterol, hypercholesterolemia, hypertriglyceridemia, and/or having been prescribed dyslipidemia therapy. T2DM was determined using the eMERGE definition developed by Northwestern University (35).

### Statistical Analysis

Statistical analysis was performed in R version 4.3.2 (R Foundation for Statistical Computing, Vienna, Austria). Continuous variables are presented as median (with 25th and 75th percentiles) and categorical variables as frequencies with percentages. Weight stable was considered the reference group in determining which weight trajectory indicator (weight cycler, weight gainer, weight loser) confers the greatest risk for each of the 10 cardiometabolic disease states. Kaplan-Meier curves were generated for each disease state to display when the onset of disease occurred and compare the cumulative incidence of disease between weight trajectory groups. Participants having the disease state prior to the landmark period were censored. Multivariate Cox proportional hazards regression models with hazard ratios adjusted for age, sex, self-reported race, self-reported ethnicity, height, baseline BMI, smoking status, and medications were developed to determine associations between weight trajectory groups and each disease state individually. Antidiabetic, antihypertensive, and antihyperlipidemia medications were included as covariates except when modeling T2DM, HTN, and HLD, respectively, due to redundancy. In addition to using linear interpolation for creating the standardized grid, all models included the number of weights recorded during the 5-year observation period to further account for differences among participants.

## Results

### Descriptive Characteristics

The final dataset, which was 60.3% female, included 83 261 unique participants with a median age of 50.5 (38.9, 61.3) years and a median BMI of 27.6 (24.1, 32.1) kg/m^2^. Among the 83,261, 61.1% had no past or current smoking usage (Table 1). During the 5-year observation period, participants had a median of 14 (10, 21) visits at VUMC with weights documented in the EHR, with cyclers having a median of 17 visits and weight-stable participants having a median of 10 visits. The median length of the landmark period was 5.2 (3.3, 8.0) years with the 99th percentile at 12.7 years of follow-up. As presented in Table 2, the cardiometabolic disease diagnoses with the highest cumulative incidence after the observation period were HDL (10.2%), HTN (8.8%), OSA (6.5%), and T2DM (5.2%).

**Table 1.** Baseline characteristics of study population by weight trajectory group

	All	Cycler	Gainer	Loser	Stable
Sample size (%)	83 261	45 294 (54.4)	19 099 (22.9)	10 997 (13.2)	7871 (9.5)
Age (years)	50.5 [38.9, 61.3]	49.4 [37.2, 60.3]	47.9 [36.9, 58.0]	56.9 [46.0, 67.2]	54.8 [44.9, 64.3]
Sex (male)	33 060 (39.7%)	16 659 (36.8%)	7631 (40.0%)	4695 (42.7%)	4075 (51.8%)
BMI (kg/m^2^)	27.6 [24.1, 32.1]	28.0 [24.2, 32.7]	26.7 [23.5, 31.0]	28.4 [24.9, 32.8]	27.0 [23.8, 30.5]
Race (self-reported) (%)					
White	72 237 (86.8)	38 841 (85.8)	16 651 (87.2)	9697 (88.2)	7048 (89.5)
Black	8773 (10.5)	5349 (11.8)	1882 (9.9)	986 (9.0)	556 (7.1)
Asian	1443 (1.7)	657 (1.5)	377 (2.0)	210 (1.9)	199 (2.5)
Other	808 (1.0)	447 (1.0)	189 (1.0)	104 (0.9)	68 (0.9)
Ethnicity (self-reported) (%)					
Hispanic/Latino	1194 (1.4)	683 (1.5)	295 (1.5)	120 (1.1)	96 (1.2)
Non-Hispanic/Latino	81 747 (98.2)	44 463 (98.2)	18 723 (98.0)	10 819 (98.4)	7742 (98.4)
Unknown	320 (0.4)	148 (0.3)	81 (0.4)	58 (0.5)	33 (0.4)
Smoking status (yes/no) (%)					
Never smoker	50 914 (61.1)	26 485 (58.5)	12 532 (65.6)	6619 (60.2)	5278 (67.1)
Ever smoker	25 043 (30.1)	14 390 (31.8)	5229 (27.4)	3354 (30.5)	2070 (26.3)
Unknown	7304 (8.8)	4419 (9.8)	1338 (7.0)	1024 (9.3)	523 (6.6)
Weights recorded (no.)	14 [10, 21]	17 [12, 25]	12 [9, 18]	13 [9, 18]	10 [7, 13]
follow-up (years)	5.2 [3.3, 8.0]	5.2 [3.2, 7.9]	5.4 [3.4, 8.2]	5.1 [3.2, 7.7]	5.4 [3.5, 8.1]

Categorical data are presented as number (percent) and continuous data are presented as median [interquartile range]. Years of follow-up is the duration of the observation period after the landmark date.

Abbreviation: BMI, body mass index.

**Table 2.** Proportion of study population with cardiometabolic outcomes by weight trajectory group

	All	Cycler	Gainer	Loser	Stable
Peripheral artery disease (%)					
Before landmark date	3133 (3.8)	2121 (4.7)	434 (2.3)	415 (3.8)	163 (2.1)
After landmark date	2151 (2.6)	1262 (2.8)	383 (2.0)	324 (2.9)	182 (2.3)
Coronary artery disease (%)					
Before landmark date	5305 (6.4)	3311 (7.3)	927 (4.9)	710 (6.5)	357 (4.5)
After landmark date	3654 (4.4)	2040 (4.5)	682 (3.6)	581 (5.3)	351 (4.5)
MASLD (%)					
Before landmark date	2026 (2.4)	1451 (3.2)	283 (1.5)	210 (1.9)	82 (1.0)
After landmark date	2447 (2.9)	1532 (3.4)	543 (2.8)	214 (1.9)	158 (2.0)
Obstructive sleep apnea (%)					
Before landmark date	8701 (10.5)	5585 (12.3)	1821 (9.5)	866 (7.9)	429 (5.5)
After landmark date	5417 (6.5)	3095 (6.8)	1347 (7.1)	567 (5.2)	408 (5.2)
Heart failure (%)					
Before landmark date	4174 (5.0)	2954 (6.5)	545 (2.9)	510 (4.6)	165 (2.1)
After landmark date	2580 (3.1)	1575 (3.5)	433 (2.3)	409 (3.7)	163 (2.1)
Myocardial infarction (%)					
Before landmark date	3544 (4.3)	2160 (4.8)	698 (3.7)	465 (4.2)	221 (2.8)
After landmark date	3147 (3.8)	1738 (3.8)	589 (3.1)	510 (4.6)	310 (3.9)
Hypertension (%)					
Before landmark date	40 246 (48.3)	22 789 (50.3)	8032 (42.1)	5907 (53.7)	3518 (44.7)
After landmark date	7336 (8.8)	3813 (8.4)	1902 (10.0)	874 (7.9)	747 (9.5)
Hyperlipidemia (%)					
Before landmark date	53 755 (64.6)	29 498 (65.1)	11 644 (61.0)	7526 (68.4)	5087 (64.6)
After landmark date	8528 (10.2)	4610 (10.2)	2181 (11.4)	962 (8.7)	775 (9.8)
Type 2 diabetes (%)					
Before landmark date	10 506 (12.6)	6536 (14.4)	1526 (8.0)	1849 (16.8)	595 (7.6)
After landmark date	4365 (5.2)	2487 (5.5)	1105 (5.8)	437 (4.0)	336 (4.3)

Abbreviation: MASLD, metabolic dysfunction-associated steatotic liver disease.

### Weight Trajectory Measures as Predictors of Cardiometabolic Diseases

**Figure 3.**
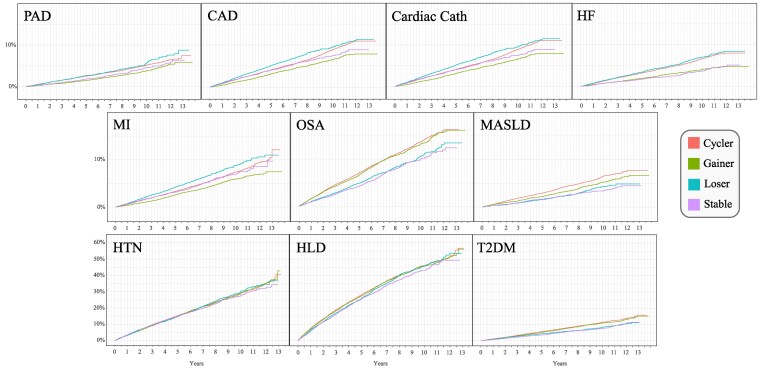
Cumulative incidence curves.

Out of 83 261 patients, 54.4% were weight cyclers, 22.9% weight gainers, 13.2% weight losers, and 9.5% weight stable. Figure 3 presents Kaplan-Meier plots comparing the cumulative incidence for each disease state by weight trajectory group. Compared to weight-stable participants, unadjusted Cox proportional hazards regression demonstrated that 1.29 times as many weight cyclers developed PAD, 1.08 as many weight cyclers developed CAD, 1.01 as many weight cyclers had cardiac catheterization, and 1.03 as many weight cyclers developed MI during the landmark period. However, no weight trajectory measure was a statistically significant predictor of increased or decreased risk for PAD, CAD, cardiac catheterization, or MI in models adjusted for age, sex, height, baseline BMI, smoking status, self-reported race and ethnicity, medications, and the number of weights recorded. As expected, age was the strongest predictor for these outcomes, and being female was associated with reduced risk.

**Figure 4.**
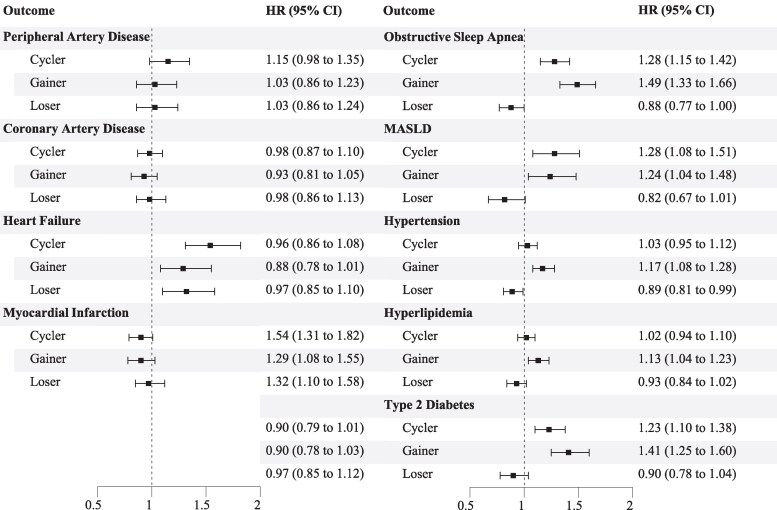
Adjusted cardiometabolic risk by weight group relative to weight stable.

However, adjusted modeling showed that weight cycling was significantly associated with a more than 50% increased risk for HF [hazard ratio (HR) 1.54; 95% confidence interval (CI) 1.31, 1.82] as well as an almost 30% increased risk for OSA (HR 1.28; 95% CI 1.15, 1.42), MASLD (HR 1.28; 95% CI 1.08, 1.51), and T2DM (HR 1.23; 95% CI 1.10, 1.38) compared to being weight stable (Fig. 3). Weight gain and weight loss were similarly associated with increased risk for HF (HR 1.29; 95% CI 1.08, 1.55 and HR 1.32; 95% CI 1.10, 1.58, respectively). Weight gain was also significantly associated with increased risk for OSA, MASLD, T2DM, HTN, and HLD (Table 3). In contrast, weight loss was significantly associated with an 11% reduced risk for HTN (Fig. 4). The number of weights recorded during the 5-year observation period significantly influenced the final model of all disease states except CAD. For each of the other disease states, any additional weight recording in the EHR resulted in a 1% increased risk.

**Table 3.** Results from multivariable Cox regression models for cardiometabolic outcomes

	Peripheral artery disease	Coronary artery disease	Heart failure	Myocardial infarction	Obstructive sleep apnea
Weight trajectory					
Cycler	1.15 (0.98, 1.35)	0.98 (0.87, 1.10)	1.54 (1.31, 1.82)	0.90 (0.79, 1.01)	1.28 (1.15, 1.42)
Gainer	1.03 (0.86, 1.23)	0.93 (0.81, 1.05)	1.29 (1.08, 1.55)	0.90 (0.78, 1.03)	1.49 (1.33, 1.66)
Loser	1.03 (0.86, 1.24)	0.98 (0.86, 1.13)	1.32 (1.10, 1.58)	0.97 (0.85, 1.12)	0.88 (0.77, 1.00)
Age	1.06 (1.05, 1.06)	1.04 (1.04, 1.04)	1.06 (1.06, 1.07)	1.03 (1.03, 1.03)	1.00 (1.00, 1.00)
Height	1.00 (0.99, 1.00)	0.99 (0.99, 1.00)	1.00 (1.00, 1.01)	0.99 (0.99, 1.00)	1.01 (1.00, 1.01)
BMI	1.00 (0.99, 1.01)	1.00 (1.00, 1.01)	1.03 (1.02, 1.03)	1.00 (1.00, 1.01)	1.08 (1.07, 1.08)
Sex (female)	0.76 (0.67, 0.87)	0.47 (0.42, 0.52)	0.82 (0.72, 0.92)	0.49 (0.44, 0.54)	0.62 (0.57, 0.67)
Race					
Asian	0.83 (0.52, 1.32)	0.72 (0.50, 1.02)	0.74 (0.47, 1.17)	0.60 (0.39, 0.92)	0.97 (0.75, 1.24)
Black	1.62 (1.43, 1.82)	1.28 (1.16, 1.41)	1.39 (1.24, 1.55)	1.08 (0.96, 1.21)	0.93 (0.86, 1.01)
Other	0.78 (0.40, 1.52)	1.14 (0.76, 1.72)	1.11 (0.67, 1.84)	1.40 (0.95, 2.07)	1.54 (1.18, 2.01)
Ethnicity					
Hispanic/Latino	0.54 (0.29, 1.00)	0.74 (0.50, 1.08)	0.36 (0.19, 0.69)	0.62 (0.40, 0.97)	0.83 (0.64, 1.07)
Unknown	0.76 (0.18, 3.12)	0.51 (0.16, 1.61)	0.43 (0.10, 1.79)	0.47 (0.12, 1.94)	0.58 (0.26, 1.31)
Smoking					
Ever	1.76 (1.61, 1.92)	1.33 (1.24, 1.42)	1.21 (1.11, 1.32)	1.27 (1.18, 1.36)	1.08 (1.02, 1.15)
Unknown	1.47 (1.22, 1.77)	1.53 (1.34, 1.76)	1.81 (1.56, 2.09)	1.21 (1.03, 1.41)	0.65 (0.56, 0.76)
Antihypertensive medication	3.15 (2.37, 4.19)	9.45 (6.61, 13.5)	32.10 (13.3, 77.3)	8.05 (5.49, 11.8)	1.45 (1.33, 1.59)
Dyslipidemia medication	2.09 (1.91, 2.29)	4.55 (4.22, 4.91)	2.04 (1.88, 2.22)	6.32 (5.78, 6.92)	1.28 (1.21, 1.36)
Antidiabetic medication	2.02 (1.83, 2.22)	1.98 (1.84, 2.13)	2.74 (2.50, 3.00)	2.05 (1.89, 2.23)	1.26 (1.19, 1.34)
Number of weights recorded	1.01 (1.01, 1.01)	1.01 (1.01, 1.01)	1.01 (1.01, 1.01)	1.01 (1.01, 1.01)	1.01 (1.01, 1.01)

	MASLD	Hypertension	Hyperlipidemia	Type 2 diabetes
Weight trajectory				
Cycler	1.28 (1.08, 1.51)	1.03 (0.95, 1.12)	1.02 (0.94, 1.10)	1.23 (1.10, 1.38)
Gainer	1.24 (1.04, 1.48)	1.17 (1.08, 1.28)	1.13 (1.04, 1.23)	1.41 (1.25, 1.60)
Loser	0.82 (0.67, 1.01)	0.89 (0.81, 0.99)	0.93 (0.84, 1.02)	0.90 (0.78, 1.04)
Age	0.99 (0.98, 0.99)	1.03 (1.03, 1.03)	1.01 (1.01, 1.01)	1.01 (1.01, 1.01)
Height	1.00 (1.00, 1.01)	1.00 (0.99, 1.00)	1.00 (1.00, 1.00)	1.00 (0.99, 1.00)
BMI	1.05 (1.05, 1.06)	1.04 (1.04, 1.04)	1.01 (1.01, 1.02)	1.08 (1.08, 1.09)
Sex (female)	0.83 (0.73, 0.93)	0.73 (0.68, 0.78)	0.88 (0.82, 0.94)	0.69 (0.63, 0.76)
Race				
Asian	1.66 (1.25, 2.20)	1.17 (0.98, 1.39)	1.32 (1.12, 1.55)	2.07 (1.67, 2.58)
Black	0.78 (0.69, 0.89)	1.41 (1.30, 1.53)	1.07 (1.00, 1.15)	1.43 (1.32, 1.55)
Other	1.38 (0.95, 2.00)	1.35 (1.06, 1.73)	1.33 (1.06, 1.68)	1.12 (0.78, 1.61)
Ethnicity				
Hispanic/Latino	1.13 (0.82, 1.57)	1.03 (0.85, 1.26)	1.06 (0.88, 1.28)	1.02 (0.77, 1.36)
Unknown	0.52 (0.13, 2.09)	0.65 (0.38, 1.12)	0.81 (0.53, 1.24)	0.91 (0.40, 2.05)
Smoking				
Ever	1.18 (1.09, 1.29)	1.19 (1.13, 1.25)	1.16 (1.11, 1.22)	1.20 (1.13, 1.28)
Unknown	0.74 (0.59, 0.94)	0.95 (0.84, 1.07)	1.04 (0.94, 1.15)	1.10 (0.95, 1.28)
Antihypertensive medication	1.91 (1.65, 2.22)	N/A	1.70 (1.62, 1.79)	3.02 (2.65, 3.44)
Dyslipidemia medication	1.33 (1.21, 1.45)	1.56 (1.48, 1.65)	N/A	1.60 (1.50, 1.71)
Antidiabetic medication	1.65 (1.51, 1.80)	1.81 (1.72, 1.90)	1.61 (1.53, 1.70)	N/A
Number of weights recorded	1.01 (1.01, 1.01)	1.02 (1.01, 1.02)	1.01 (1.01, 1.01)	1.01 (1.00, 1.01)

Data are presented as odds ratio (95% confidence interval). Reference for weight behavior is weight-stable group, for sex is female, for race is White, for ethnicity is Non-Hispanic/Latino, for smoking is never smokers, and for medications is no record of medication use.

Abbreviations: BMI, body mass index; MASLD, metabolic dysfunction-associated steatotic liver disease.

## Discussion

In this large regional medical center cohort of >83 000 adult patients, who had a prevalence of weight cycling of 54% and median follow-up of 5.2 years, we observed that independent of other typical demographic and clinical biomarkers, being a weight cycler significantly increased risk for HF, OSA, MASLD, and T2DM when compared to weight stability. It must be recognized that because baseline BMI was similar among the weight trajectory groups, with both median and mean baseline weight for height in the overweight BMI category, the impact of weight cycling on the risk for developing these highly burdensome cardiometabolic disease states is not merely driven by high body mass.

Recent evidence from preclinical models shows that the chronic inflammation induced by high adiposity is driven by proinflammatory immune cells such as CD8^+^T cells and lipid associated macrophages that not only infiltrate metabolically active tissues but create an obesogenic “memory” that persists after weight loss and consequently exacerbates the degree of cardiometabolic dysfunction associated with episodes of weight cycling (18, 36, 37). Effects of this condition of ongoing antagonistic inflammation in adipose tissue include insulin resistance, the development of atherosclerosis, and proinflammatory changes in epicardial adipose tissue that promote increased risk for HF.

It is interesting that we observed weight cycling, weight gain, and weight loss increased the risk for HF when compared to weight stability, despite no significant differences in baseline BMI among the groups. Notably, twice as many weight cyclers were diagnosed with HF during the landmark period. Yet, no significant association was detected between weight trajectory group and CAD or MI, the most common etiologies of HF (38). This contrast with other findings may be due to most studies of weight cycling grouping HF, CAD, and MI as a composite outcome of cardiovascular disease (7, 40, 41). Also intriguing is that while weight loss associated with increased HF risk, it also associated with a substantially decreased risk for HTN, another common cause of HF (39). Taken together, these findings suggest the bidirectionality of weight trajectory and HF diagnosis—significant weight change may be a marker of subclinical HF or having HF may induce weight change. Consideration might be given to the utility of weight trajectory as an early screening tool in the prehospital environment where HF is commonly underdiagnosed (42).

Weight cyclers showed the highest risk for PAD, MASLD, and HF when compared to the weight-stable group. It is notable that weight cyclers were associated with a greater risk for cardiovascular-related disease states typically diagnosed via routine screening methods rather than definitive events such as MI. A potential explanation for this unexpected finding may be the lengthy natural history of atherosclerotic disorders. Although plaque formation may begin in childhood, symptomatic rupture and occlusive lesions typically occur decades later if at all (43). Thus, it is plausible that the landmark follow-up period was inadequate to capture relationships between weight cycling and atherosclerotic disease.

In contrast, weight gainers showed a greater risk for OSA, HTN, HLD, and T2DM. The role of body weight, particularly excess body weight, is well established in these 4 disease states, although studies investigating the temporality of relationships between weight trajectory and incidence of disease are limited. One prospective cohort of 690 adults showed a 5% increase in weight over 4 years was associated with a 15% increased incidence of moderate to severe OSA as compared to weight-stable persons with similar high baseline BMI (44). Population-based cohort studies investigating the long-term impact of weight change have also shown a 6% increase in weight over 5 to 6 years increased the risk for HTN 2-fold (45, 46). Likewise, weight gain ≥5% over 6 years increased the risk for T2DM by 76% (47).

The specific mechanisms whereby a change in body weight directly affects cardiometabolic risk require further exploration. The composition of typical weight gain is ∼70% adipose tissue. Studies from bariatric surgery reveal a key role for signaling of endocrine hormones that are secreted in adipose tissues (48). Other studies suggest roles for macrophage recruitment, inflammation, and oxidative stress in adipose tissues—all of which can be reversed with sustained weight loss. In contrast to weight gain, weight loss ≥5% was associated with reducing the incidence of HTN by 11% as well as decreasing risk for OSA by 12% when compared to weight stability. Yet only 13.2% of the cohort were continuous weight losers. Unexpectedly, the amount of weight loss, perhaps due to the older age of the weight loser group, prevented observation of statistically significant associations between weight loss and other cardiometabolic disease states typically benefited by losing weight, including MASLD, HLD, and T2DM.

The findings from this investigation must be interpreted considering the limitations and strengths of the study. Although patients with a diagnosis of cancer were excluded, it is not possible using EHR data to eliminate all bias due to intentional vs unintentional weight loss. Also noteworthy is that the number of weights recorded during the observation period was a significant predictor for all disease outcomes except CAD. This may reflect that patients with more visits where weights are documented are less healthy or more health-seeking and therefore more likely to have coded diagnoses, providing greater opportunity to capture weight-cycling events (19). These data demonstrate that accounting for the number of recorded weights (or other similar vital sign type variables) both statistically and in study design is critical for future studies investigating outcomes using retrospective EHR data. Further, key factors that affect cardiometabolic health such as dietary intake, physical activity, and socioeconomic status are typically not available in the EHR. Similarly, actual smoking, vaping, alcohol, cannabis, and narcotics use are most often inadequately captured in the EHR. Moreover, some medications, such as therapies for dyslipidemia, may have been prescribed for other high-risk comorbidities (eg, diabetes or stroke) without a patient having elevated blood lipids. Additionally, EHR data may possess other intrinsic limitations that may not be detected, such as data entry errors. Due to the nature of this dataset, an extensive multiphase data cleaning process was incorporated to ensure data reliability. The use of linear interpolation to standardize weights also reduced error. Other strengths of the study include the large sample size and the acquisition of data from a comprehensive regional healthcare system that comprises 7 hospitals and >180 patient clinics.

## Conclusions

In this cohort of >80 000 community residing adults, over half experienced at least 1 full episode of weight cycling within a 5-year observation period that included a median of 14 visits to the medical center. We found that weight cycling significantly increased the risk for incident HF, OSA, MASLD, and T2DM when compared to weight stability—independent of baseline BMI as well as other traditional clinical risk factors. Since weight gain also demonstrates great risk, the present findings support the promotion of either weight stability at higher BMI or weight loss if able to be maintained as critical strategies to prevent the incidence of a variety of cardiometabolic diseases. Such a paradigm shift would align with achieving more personalized care and providing targeted interventions to the people who would most benefit. Furthermore, greater public health efforts are required to publicize the importance of minimizing changes in body weight over time when weight loss cannot be maintained.

## Data Availability

Some or all datasets generated during and/or analyzed during the current study are not publicly available but are available from the corresponding author on reasonable request.
